# Multi-step forecasting of dissolved oxygen in River Ganga based on CEEMDAN-AdaBoost-BiLSTM-LSTM model

**DOI:** 10.1038/s41598-024-61910-w

**Published:** 2024-05-16

**Authors:** Neha Pant, Durga Toshniwal, Bhola Ram Gurjar

**Affiliations:** 1 Computer Science and Engineering, Indian Institute of Technology Roorkee, Roorkee, 247667 India; 2 Civil Engineering, Indian Institute of Technology Roorkee, Roorkee, 247667 India

**Keywords:** Complete ensemble empirical mode decomposition with adaptive noise, AdaBoost, Long short-term memory, Water quality forecasting, Environmental sciences, Hydrology

## Abstract

Accurate prediction of Dissolved Oxygen (DO) is an integral part of water resource management. This study proposes a novel approach combining Complete Ensemble Empirical Mode Decomposition with Adaptive Noise (CEEMDAN) with AdaBoost and deep learning for multi-step forecasting of DO. CEEMDAN generates Intrinsic Mode Functions (IMFs) with different frequencies, capturing non-linear and non-stationary characteristics of the data. The high-frequency and medium-frequency IMFs, characterized by complex patterns and frequent changes over time, are predicted using Adaboost with Bidirectional Long Short-Term Memory (BiLSTM) as the base estimator. The low-frequency IMFs, characterized by relatively simple patterns, are predicted using standalone Long Short-Term Memory (LSTM). The proposed CEEMDAN-AdaBoost-BiLSTM-LSTM model is tested on data from ten stations of river Ganga. We compare the results with six models without decomposition and four models utilizing decomposition. Experimental results show that using a tailored prediction technique based on each IMF’s distinctive features leads to more accurate forecasts. CEEMDAN-AdaBoost-BiLSTM-LSTM outperforms CEEMDAN-BiLSTM with an average improvement of 25.458% for RMSE and 37.390% for MAE. Compared with CEEMDAN-AdaBoost-BiLSTM, an average improvement of 20.779% for RMSE and 28.921% for MAE is observed. Diebold-Mariano test and t-test suggest a statistically significant difference in performance between the proposed and compared models.

## Introduction

Water is an extremely significant and indispensable asset on our planet, necessary for the existence of human beings, wildlife, and vegetation. Dissolved oxygen (DO) is an important quality parameter that has been used in numerous studies to assess the standard of water quality in aquatic ecosystems^1–3^. Low levels of DO indicate poor water quality, which can be harmful to aquatic life whereas high quantities usually suggest a healthy and well-oxygenated aquatic ecosystem. Accurate DO forecasting is critical for successful water resource management as it allows authorities to foresee and respond to changes in water quality.

Classic statistical models like Multiple Linear Regression(MLR)^4^, autoregressive moving average (ARMA)^5^ and autoregressive integrated moving average (ARIMA)^6^ are designed primarily for linear relationships and can find it difficult to capture and model the non-linear and non-stationary patterns present in the water quality time series data. In recent years machine learning and deep learning models have increasingly been used for forecasting which can be attributed to a multitude of factors, encompassing the availability of extensive datasets, advancements in computational resources, breakthroughs in neural network architectures, and notable algorithmic advancements. A study by Sahoo et al.^7^ investigates the applicability of Support Vector Regression (SVR) in modeling monthly low flows hydrological time series. To forecast Chlorophyll a, Liang et al.^8^ trained a variety of Long Short-Term Memory (LSTM) with different combinations of input variables, hidden layer numbers, and lag periods. Sahoo et al.^9^ uses Convolutional Neural Network combined with bi-directional LSTM for forecasting of urban water demand. Zou et al.^10^ put forth a recommendation for the prediction of water quality in the Beilun River by employing a multi-time scale bidirectional LSTM approach. Bi et al.^11^ offers a hybrid model based on a LSTM-based encoder-decoder network and a Savitzky-Golay filter. Huang et al.^12^ introduced an approach for predicting water quality utilizing a combination of k-nearest-neighbor probability rough sets and PSO-LSTM. The accuracy of a artificial intelligence models can be greatly enhanced by boosting and can aid in improving generalization and reducing overfitting. In the study by Aldrees et al.^13^, boosting and bagging ensemble models are compared with individual network-based and tree-based models for making predictions. El Bilali et al.^14^ discussed the applicability of using AdaBoost and Random Forest for predicting groundwater quality.

Often water quality time series data exhibits large fluctuations, non-linearity, and non-stationary characteristics^15^ which makes the task of forecasting challenging. To address this, pre-processing the data using signal decomposition techniques can be useful in transforming the complex dataset into relatively simple sub-series. Sahoo et al.^16^, a uses Fourier Transform combined with LSTM to predict daily suspended sediment load. Huang et al.^17^ introduced Empirical Mode Decomposition (EMD) which is a signal processing technique that analyzes non-stationary signals by breaking them into Intrinsic Mode Functions (IMFs) according to their local characteristics. Wu et al.^18^ proposed the Ensemble Empirical Mode Decomposition (EEMD) technique to overcome the limitations of EMD by reducing the effects of noise and mode mixing in the decomposition process. However, EEMD has some drawbacks, such as increased computational complexity and the possibility of over-smoothing of the IMFs. Complete Ensemble Empirical Mode Decomposition with Adaptive Noise (CEEMDAN) technique^19^ was introduced as an improvement to EEMD, where a fixed noise level is added to the signal to generate the ensemble realizations, CEEMDAN employs a noise level that incrementally grows and adjusts according to the specific characteristics of the local signal. CEEMDAN has another advantage over EEMD in that it is more effective at addressing the issue of mode mixing. Zhang and Yang^20^ coupled CEEMDAN with Gated recurrent units (GRU) to forecast suspended sediment concentration in the Yellow River and outperformed Support Vector Machines, LSTM and GRU standalone models. Lu and Ma^21^ suggested using CEEMDAN approach with XGBoost and Random Forest. Zhang et al.^22^ suggested the decomposition of water quality data into IMFs using CEEMDAN algorithm and combining it with LSTM model for forecasting the water quality. Song and Yao^23^ suggests using CEEMDAN decomposition in conjunction with LSTM for water quality prediction.^24^ recommends using CEEMDAN and Variational Mode Decomposition (VMD) combined with Least Square Support Vector Machine (LSSVM) and Extreme Learning Machines (ELM) for estimating water quality parameters.

The ability of CEEMDAN to decompose the time series into IMFs facilitates the separation and analysis of various frequency components. This can help to detect the non-linear and non-stationary properties of the data. Deep learning and machine learning models have the capacity to learn and represent complex relationships between input variables, enabling them to capture both linear and non-linear dependencies in the data. This is especially useful in forecasting water quality since the interactions between input factors and water quality metrics can be complicated and multifaceted. This motivated us to investigate the potential of CEEMDAN decomposition in combination with AdaBoost and deep learning models in order to capture the long-term relationships accurately for DO forecasting. First, the CEEMDAN technique is used to decompose the data into a set of IMF sub-series components. The high and medium-frequency sub-series are forecasted using the Adaboost-BiLSTM, while the low-frequency components are forecasted using the standalone LSTM model. For a comprehensive analysis, the CEEMDAN-AdaBoost-BiLSTM-LSTM model is evaluated using data collected from ten different stations of river Ganga and compared with six standalone models and four models utilizing decomposition. Diebold Mariano test and two-sided t-test further suggests that there is a statistically significant difference in the forecasting performance of our proposed model as compared to the alternative models.

## Methods

### Study area and data collection

River Ganga, also known as the Ganges, is a transboundary river that flows through India and Bangladesh. It begins in the Himalayas, travels about 2525 kilometers, and empties into the Bay of Bengal. Apart from its cultural and spiritual significance, the Ganga River enables inland navigation and supports key sectors like as agriculture, fishing, tourism, and hydropower generation, all of which contribute to the local economy. Unfortunately, the water in the Ganga river is severely polluted, and the primary sources of which can be attributed to the unregulated release of untreated wastewater, industrial waste, and the inflow of agricultural runoff^25,26^.

**Figure 1:**
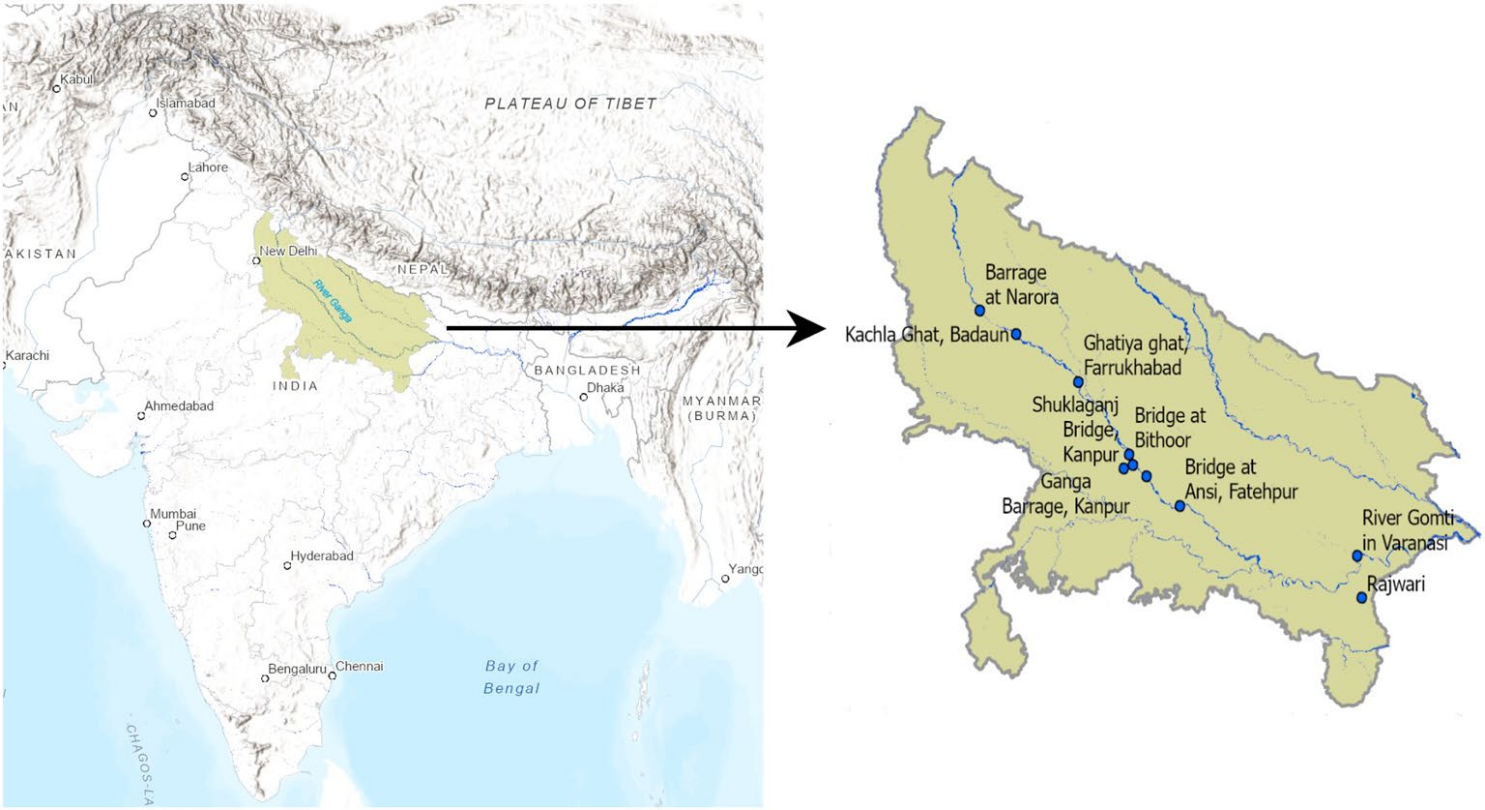
The geographic locations of the ten stations of River Ganga in Uttar Pradesh, India are denoted by blue markers.

River Ganga has a length of around 1450 kilometers in Uttar Pradesh. The data used in this study was collected from the Uttar Pradesh Pollution Control Board, Government of India. This dataset comprises of real-time measurements of DO levels obtained through the Real-time Water Quality Monitoring System installed at multiple locations along the River Ganga, its principal tributaries, and drains. The water quality data utilised in this study is for ten stations of River Ganga and its tributaries in Uttar Pradesh and spans from 1 April 2017 to 30 September 2021. In the original dataset, the sampling frequency ranged from one sample per hour for the years 2017 and 2018 to one sample every 15 min from the year 2019 to 2021. The specific locations analyzed in this study are distinctly marked and shown in Fig. 1. More details about the data considered in the study are provided in Supplementary Information and Supplementary Table S1.

### CEEMDAN

CEEMDAN^19^ uses an adaptive noise level, which adjusts the noise level of each ensemble realization based on the local signal characteristics, to enhance decomposition performance and efficiency. For the CEEMDAN decomposition process, first, we add white noise to the original time series signal X(t). This can be expressed as follows:

1 $$\begin{aligned} X^i(t)= X(t) + s_{0}\omega ^i(t) \end{aligned}$$

where $s_0$ is the noise coefficient for controlling the signal-to-noise ratio and $\omega ^i(t)$ denotes the $i^{th}$ noise for $i = 1... N$, where *N* is the number of times EMD is performed. We use EMD to decompose each $X^i(t)$ into $IMF^i(t)$. The first IMF component can be obtained by averaging all the modes. This can be mathematically expressed as follows:

2 $$\begin{aligned} \overline{IMF_1(t)} = \frac{1}{N} \sum _{i=1}^{N}{IMF_1^i(t)} \end{aligned}$$

The initial residual is computed by subtracting the first IMF from the original signal as follows:

3 $$\begin{aligned} r_1(t) = X(t) - \overline{IMF_1(t)} \end{aligned}$$

The rest of the IMFs and residual can be calculated as follows:

4 $$\begin{aligned} \overline{IMF_k(t)} = \frac{1}{N} \sum _{i=1}^{N}{E_{k-1}(r_{k-1}(t) + E_{k-1}(p_{k-1}\omega ^i(t)))} \end{aligned}$$

5 $$\begin{aligned} r_k(t) = r_{k-1}(t) - \overline{IMF_k(t)} \end{aligned}$$

where, $E_k(.)$ extracts the $k^{th}$ IMF that EMD has decomposed. The calculation of the final residual involves subtracting the sum of all the *K* IMFs from the original signal, resulting in the following expression

6 $$\begin{aligned} R(t) = X(t) - \sum _{k=1}^{K}\overline{IMF_k(t)} \end{aligned}$$

### AdaBoost

AdaBoost is a machine learning algorithm developed by by Freund et al.^27^. It employs a combination of weak classifiers to construct a strong classifier with enhanced performance. Although it was originally designed for classification problems, it can also be adapted for regression tasks^28^. In AdaBoost regression, weak learners are replaced with regression models that can predict continuous values. The algorithm iteratively fits regression models to the data and assigns weights to the samples based on their error. More weight is given to poorly predicted samples in subsequent iterations. The final prediction is a weighted combination of all the regression models’ predictions, with the weights determined by their performance on the training data. AdaBoost uses the following steps for the computation:

Let the data points used for training be $(x_1,y_1)$ ... $(x_N,y_N)$.

- *Step 1* Initialize the number of iterations as T. Let for the initial iteration, $t = 1$ and average loss $\overline{L_t} = 0$. Initialize the sample weights distribution as $D_t(i) = \frac{1}{N}$ for $i = 1...N$
- *Step 2* Using the sample weights, train the regression model. Let $f(x_i)$ be the predicted value for the $i^{th}$ sample.
- *Step 3* For each training sample compute the loss as : $l_t(i) = \vert f(x_i) - y_i \vert$
- *Step 4* The loss function for each individual training instance can be calculated as:
  7 $$\begin{aligned} L_t(i) =l_t(i)/M_t \end{aligned}$$
  where, $$M_t = max_{i=1}^{N}l_t(i)$$
- *Step 5* Compute the average loss: $\overline{L_t} = \sum _{i=1}^{N} L_t(i)D_t(i)$
- *Step 6* Set $$\beta _t = \overline{L_t}/(1-\overline{L_t})$$
- *Step 7* Now, update the weight distribution of all the samples as follows :
  8 $$\begin{aligned} D_{t+1}(i) = \frac{D_{t}(i)\beta _t^{1-L_t(i)}}{Z_t} \end{aligned}$$
  Here, $Z_t$ is the normalization factor.
- *Step 8* Now update $t = t+1$ and repeat steps 2 to 8 while t $\le$ T and average loss function $\overline{L_t} \le 0.5$
- *Step 9* Finally, the output can be given as follows:
  9 $$\begin{aligned} f_{fin}(x) = inf \left[ y \epsilon Y: {\sum _{t: f_t(x)<=y}log \left(\frac{1}{\beta _t} \right) \ge \sum _{t} \frac{1}{2} log \left(\frac{1}{\beta _t} \right)}\right] \end{aligned}$$

### LSTM

LSTM^29^ are a special type of recurrent neural network with the capacity to learn long-term dependencies by employing cell states to store information about various time periods. A individual LSTM unit’s cell state describes the data that has been thought to be relevant up to that date. By using input, output, and forget gates, LSTMs control the information flow to the cell states. The input gate assesses the importance of incoming data and decides how much of it should be retained in the memory cell. The forget gate regulates which past information should be ignored, while the output gate guarantees that only relevant information is produced at each time step. The input at the current time step, denoted as $x_t$, and the previous hidden state, denoted as $h_{t-1}$, are used to calculate the values for the input gate, output gate, and forget gate. These gate values are then utilized to update the cell state $c_t$ of the LSTM. The output gate is applied to the hyperbolic tangent of the cell state $c_t$ to produce the final hidden state $h_{t}$. The equations describing the working of LSTM are as follows:

10 $$\begin{aligned} f_{t}= & {} \sigma (U_{f}x_{t} + W_{f}h_{t-1} + b_{f}) \end{aligned}$$

11 $$\begin{aligned} i_{t}= & {} \sigma (U_{i}x_{t} + W_{i}h_{t-1} + b_i) \end{aligned}$$

12 $$\begin{aligned} o_{t}= & {} \sigma (U_{o}x_{t} + W_{o}h_{t-1} + b_o) \end{aligned}$$

13 $$\begin{aligned} c_t= & {} (f_t \times c_{t-1})+ i_t \times tanh(U_{c}x_{t} + W_{c}h_{t-1} + b_c) \end{aligned}$$

14 $$\begin{aligned} h_t= & {} o_t \times tanh(c_t) \end{aligned}$$

where, $\sigma$ represents the sigmoid function, *tanh* denotes the hyperbolic tangent function and $\times$ indicates the element-wise multiplication. $W_f, W_i, W_o, W_c$ are the input weight matrices and $U_f, U_i, U_o, U_c$ are the recurrent weight matrices for the forget, input, output and memory cell gate respectively. The bias vectors to the respective gates are represented by $b_f, b_i, b_o$ and $b_c$. The hidden state and input at timestamp *t* are denoted by $h_t$ and $x_t$ respectively.

### BiLSTM

The BiLSTM layers are comprised of a pair of LSTM layers with one of them handling the input sequence in a forward direction and the other one processing it in reverse direction. BiLSTM can capture dependencies and patterns that a unidirectional LSTM would miss by analysing the input sequence in both directions resulting in a more complete contextual understanding. The equations describing the working of BiLSTM can be expressed mathematically as below:

15 $$\begin{aligned} h_{ft}= & {} \phi (W_{fx}x_{t} + W_{fh}h_{f(t-1)} + b_{fb}) \end{aligned}$$

16 $$\begin{aligned} h_{bt}= & {} \phi (W_{bx}x_{t} + W_{bh}h_{b(t-1)} + b_{b}) \end{aligned}$$

17 $$\begin{aligned} \hat{y_t}= & {} \sigma (W_{fy}h_{ft} + W_{by}h_{bt} + b_{y}) \end{aligned}$$

here, $W_{fx}$ and $W_{bx}$ are the weight matrices from the input to the recurrent units, and $W_{fh}$ and $W_{bh}$ are the weight matrices from the recurrent units to themselves for the forward and backward layers respectively. The biases for the forward and backward layers are given by $b_{fb}$ and $b_{b}$. $\phi$ is the activation function at the hidden layers. $W_{fy}$ and $W_{by}$ are the weight matrices, and $b_{y}$ denotes the bias for the output layer.

### Proposed approach

**Figure 2:**
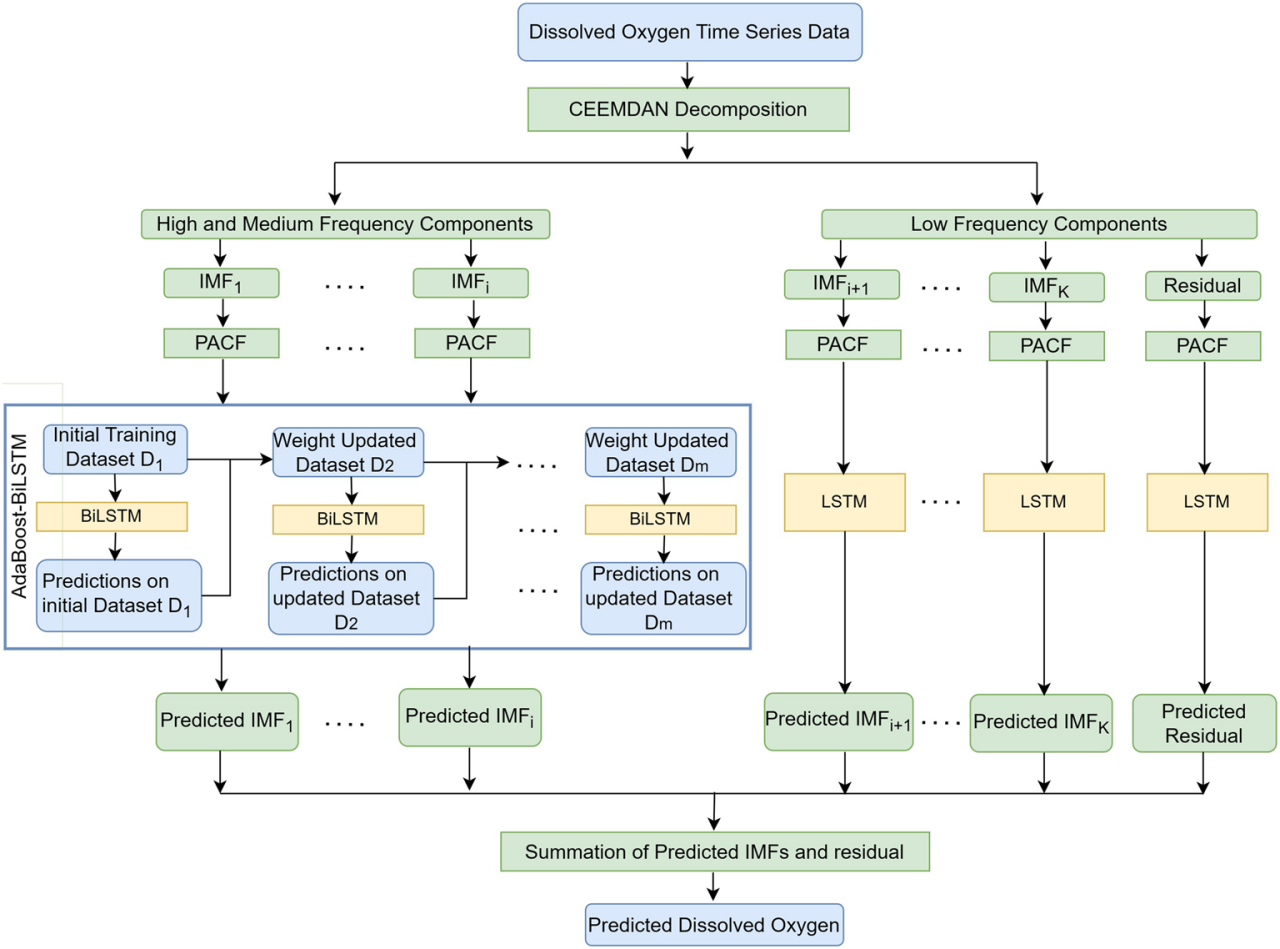
Flowchart of the proposed approach.

The steps for developing the model based on CEEMDAN decomposition combined with AdaBoost and deep learning are given below and the flowchart of the proposed model is shown in Fig. 2.

1. The first step is to use linear interpolation to fill in the missing values. The data for all the years are combined. To ensure consistent sampling frequency across the dataset, the data was extracted at a constant sample rate of one observation per hour for all years from the combined dataset.
2. The CEEMDAN approach is used to decompose the DO time series data into several IMFs and residual with each generated IMF possessing unique and distinctive inherent properties.
3. The data is partitioned into training and testing sets with a ratio of 75:25. 10$\%$ of the training data is taken for validation.
4. The data is normalized using min-max normalization, which scales the values to the [0,1] range as follows:
  18 $$\begin{aligned} x_{n} = \frac{ x - x_{min}}{x_{max}-x_{min}} \end{aligned}$$
  where, $x_{n}$ corresponds to normalized value of *x*. $x_{min}$ and $x_{max}$ denotes the minimum and maximum value of the variable.
5. For each IMF, Autocorrelation Function (ACF) and Partial Autocorrelation Function (PACF) are utilized to choose significant lagged data for constructing the forecasting models. Sliding window based multiple input multiple output approach is used to generate a collection of input-output pairs from time series data, with an input window providing historical information while the output window representing the target values.
6. The zero-crossing rate is calculated to check the fluctuation frequency of the data in each IMF and then categorize them into high, medium and low frequency components. If zero-crossing rate of IMF $\ge$ 0.01 then the IMF is categorized as having high/medium frequency otherwise it has low frequency^30^. In order to forecast high and medium frequency IMFs, the AdaBoost-BiLSTM model is utilized due to its ability to predict complex non-linear patterns. BiLSTM can handle complex sequential data and detect long-term relationships between inputs and outputs, and AdaBoost can improve prediction accuracy by combining several BiLSTMs. LSTM models are employed for the low-frequency IMFs and residual that display reasonably simple and smooth patterns. In the end, the final prediction is obtained by summing the predicted outcomes of all the IMFs and residual.

### Implementation details

The implemented model is developed using Python version 3.7. The CEEMDAN method is used with the help of EMD-signal 1.2.2 package. While implementing CEEMDAN method, the parameter settings include 100 trials and a scale of 0.05 for added noise. The number of lags needed as input to the forecasting algorithms is determined based on ACF and PACF. Supplementary Table S2 presents the identified significant time lags for the decomposed components of the time series data across ten stations. Each dataset may produce different number of IMFs on decomposition. Here, ’NA’ as the table entry represents the absence of IMF for the particular station. Optimal hyperparameters are selected while building the models (details in Supplementary Information). The Supplementary Table S3 lists the search space explored for identifying the optimal hyperparameters. The zero-crossing rate of the IMFs for all the station datasets considered are given in Supplementary Table S4. The deep learning models were implemented using TensorFlow 2.0.0 and Keras 2.3.1 packages. The number of hidden layers range from 3 to 5 and the number of neurons in the hidden layers is varied in the range of 8 to 64. To improve the gradient descent technique, the Adam optimizer was utilized. The *ReLU* activation function is used in all the hidden layers of the models. The model is trained using the learning rate of 0.001. The batch size used for training is set to 64, and a variable number of epochs ranging from 10 to 100 are employed for each IMF. Early stopping is used to prevent overfitting of the model during training.

### Performance evaluation metrics

The criteria to assess the model’s performance are Mean Absolute Error (MAE), Mean Absolute Percentage Error (MAPE), Root Mean Square Error (RMSE) and Coefficient of Determination $$($$ $$R^2$$ $$)$$ (details in Supplementary Information), mathematically, expressed as follows:

19 $$\begin{aligned} RMSE= & {} {\sqrt{\frac{\sum _{t=1}^{n} (\hat{y}_t-y_t )^2 }{n}}} \end{aligned}$$

20 $$\begin{aligned} MAE= & {} \frac{1}{n} \sum _{t=1}^{n} |\hat{y}_t-y_t| \end{aligned}$$

21 $$\begin{aligned} MAPE= & {} \frac{1}{n} \sum _{t=1}^{n} \frac{|y_t - \hat{y}_t|}{|y_t|} \times 100 \end{aligned}$$

22 $$\begin{aligned} R^2= & {} 1- \frac{\sum _{t=1}^{n} (y_t - \hat{y}_t)^2)}{\sum _{t=1}^{n} ( \overline{y} - {y}_t)^2)} \end{aligned}$$

where, *n* is the total count of the samples, $\overline{y}$ is the mean value, $y_t$ is the true value and $\hat{y}_t$ is the predicted value at time *t*.

## Results

**Figure 3:**
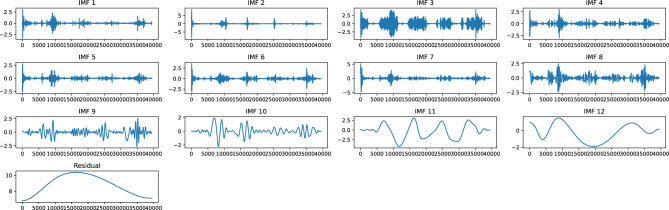
IMFs and residual generated after performing CEEMDAN decomposition of the time series data at Bithoor station.

Figure 3 shows the IMFs and residual that were generated using CEEMDAN approach for the DO time series data of Bithoor station. The time series is decomposed into 12 IMFs and a residual subseries. It can be seen that the frequency of first IMF is the highest, and as we progress toward the subsequent IMFs, the frequency gradually decreases. Based on zero-crossing rate, high and medium frequency IMFs are from IMF1 to IMF7, whereas IMF8 to IMF12 and residual are low frequency components.

**Figure 4:**
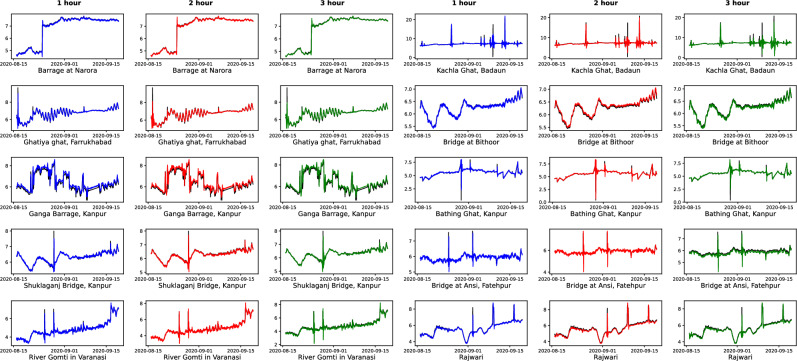
A visual representation of actual and predicted DO values over the test data at different stations.

**Figure 5:**
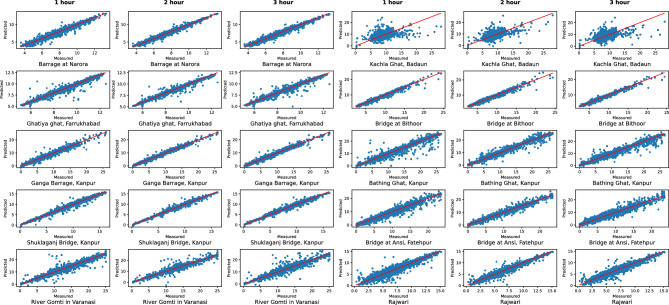
Scatter plot illustrating the alignment between observed and predicted DO values on test data.

**Figure 6:**
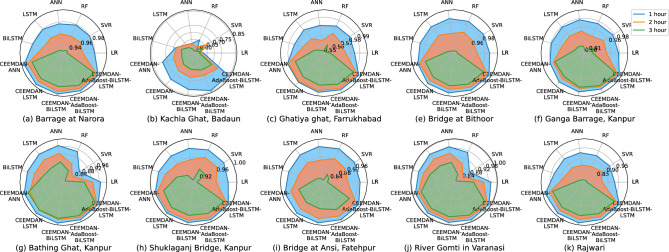
Comparison of coefficient of determination values for different models over test data across all stations.

**Figure 7:**
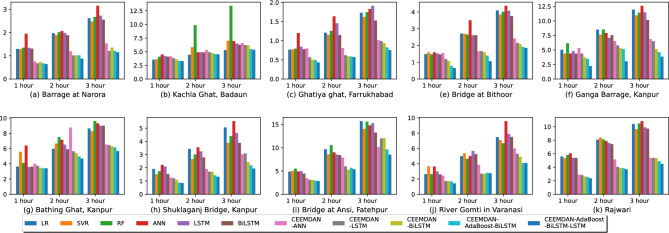
Comparison of MAPE values for different models over test data across all stations.

We have used six single models without decomposition: Linear Regression (LR), SVR, Random Forest (RF), ANN, LSTM and BiLSTM. The models utilizing decomposition are: CEEMDAN-ANN, CEEMDAN-LSTM, CEEMDAN-BiLSTM, CEEMDAN-AdaBoost-BiLSTM and CEEMDAN-AdaBoost-BiLSTM-LSTM. The observed and forecasted DO values for all stations for the test data are presented in Fig. 4. The actual values are shown in black color, and blue, red, and green lines represent the predicted values for one, two and three-hour ahead forecast, respectively. Similarly, Fig. 5 illustrates the scatter plots showcasing the relationship between the observed and predicted DO values on test data. This corresponds to higher correlation between the measured and the predicted values. Tables 1 and 2 illustrates the comprehensive comparison of the individual models and hybrid models utilizing decomposition, respectively, highlighting their performance based on RMSE and MAE values. Figure 6 displays spider plots that compare the coefficient of determination values and help to visualize the significant improvement in the performance of CEEMDAN-AdaBoost-BiLSTM-LSTM over individual models for various stations. Figure 7 illustrates the MAPE values comparing all methods with the proposed approach for all horizons for every station. It can be seen that the MAPE value for the proposed approach is lower than all other models. This demonstrates that the proposed approach has the potential to serve as an efficient means of generating dependable and accurate forecasting.

## Discussion

Among the individual models, it can be observed that the LR model gives satisfactory performance in several cases. However, it is important to note that, LR is more capable to capture linear relationships within the data. On the other hand, SVR and RF are capable of handling the non-linear and complex relationships. The ANN and deep learning models like LSTM and BiLSTM are proficient in capturing non-linear and long-term temporal dependencies in DO time series data. In terms of all evaluation metrics, the BiLSTM model consistently demonstrates superior forecasting accuracy across all forecasting horizons.

By utilizing the CEEMDAN technique to decompose the water quality data into various frequency segments and subsequently applying the deep learning models to capture the temporal dependencies within each frequency segments, a substantial improvement in forecasting accuracy has been observed as compared to the models only utilizing standalone deep learning model. The effectiveness of the CEEMDAN decomposition method in improving the precision of water quality forecasts is evident on comparing the RMSE and MAE values. When compared to standalone ANN, the CEEMDAN-ANN demonstrates better performance by achieving lower RMSE values. The reductions vary from 8.849% to 55.656%, 10.954% to 51.213%, and 7.374% to 48.359% across all stations for one, two, and three-hour ahead forecasting horizons, respectively. In terms of MAE, the reductions vary from 2.109% to 60.811%, 0.414% to 52.290%, and 10.782% to 51.440% for one-hour, two-hour, and three-hour ahead forecasts. Likewise, for CEEMDAN-LSTM model, the reductions in RMSE range from 25.556% to 54.229%, 13.610% to 50%, and 7.043% to 49.315%, for one-hour, two-hour, and three-hour ahead forecasts respectively, compared to the simple LSTM model. In terms of MAE, the reductions range from 5.063% to 45.544% for the one-hour ahead forecasts, 7.143% to 58.871% for the two-hour ahead forecasts, and 4.640% to 49.514% for the three-hour ahead forecasts. The CEEMDAN-BiLSTM exhibits superior performance by giving lower RMSE values compared to the simple BiLSTM, with reductions ranging from 22.661% to 54.271%, 7.335% to 52.877% and 3.895% to 48.732% for one, two, and three-hour ahead predictions across stations. Likewise, for MAE, reductions in the range of 1.357% to 47.535%, 3.488% to 49.484%, and 3.325% to 48.979% are observed for one-hour, two-hour, and three-hour forecasts, respectively. These results indicate that the approaches using decomposition as a data preprocessing step has a substantial influence on the efficacy of models.

**Table 1:** Prediction results on test data for single models without decomposition.

Station	Hour	LR		SVR		RF		ANN		LSTM		BiLSTM	
		RMSE	MAE	RMSE	MAE	RMSE	MAE	RMSE	MAE	RMSE	MAE	RMSE	MAE
Barrage at Narora	1	0.202	0.099	0.201	0.098	0.207	0.101	0.221	0.148	0.201	0.101	0.199	0.098
	2	0.284	0.154	0.282	0.148	0.291	0.156	0.286	0.162	0.284	0.153	0.278	0.148
	3	0.365	0.207	0.36	0.196	0.37	0.21	0.372	0.243	0.365	0.206	0.355	0.196
Kachla Ghat, Badaun	1	1.055	0.281	1.113	0.293	1.008	0.281	1.056	0.339	1.042	0.295	0.962	0.28
	2	1.086	0.327	0.997	0.362	1.086	0.357	1.059	0.369	1.058	0.364	0.968	0.344
	3	1.094	0.375	1.046	0.436	1.129	0.422	1.112	0.473	1.079	0.431	1.027	0.421
Ghatiya ghat, Farrukhabad	1	0.173	0.064	0.169	0.064	0.179	0.065	0.188	0.098	0.174	0.071	0.169	0.064
	2	0.221	0.102	0.229	0.097	0.218	0.103	0.257	0.138	0.231	0.124	0.218	0.097
	3	0.281	0.146	0.298	0.137	0.27	0.144	0.296	0.156	0.29	0.164	0.267	0.128
Bridge at Bithoor	1	0.233	0.111	0.239	0.116	0.238	0.108	0.24	0.12	0.239	0.116	0.232	0.11
	2	0.386	0.205	0.394	0.2	0.389	0.195	0.416	0.262	0.379	0.195	0.374	0.194
	3	0.547	0.312	0.549	0.288	0.555	0.294	0.553	0.331	0.552	0.317	0.529	0.284
Ganga Barrage, Kanpur	1	0.452	0.245	0.456	0.23	0.49	0.254	0.452	0.237	0.45	0.237	0.444	0.228
	2	0.677	0.402	0.667	0.366	0.737	0.41	0.676	0.39	0.669	0.37	0.666	0.361
	3	0.869	0.543	0.854	0.488	0.946	0.552	0.883	0.56	0.861	0.526	0.846	0.488
Bathing Ghat, Kanpur	1	0.738	0.227	1.45	0.438	0.931	0.286	0.764	0.365	0.756	0.24	0.738	0.217
	2	1.104	0.409	1.682	0.536	1.422	0.489	1.124	0.483	1.143	0.422	1.103	0.407
	3	1.399	0.576	1.887	0.654	1.639	0.63	1.408	0.619	1.466	0.608	1.388	0.536
Shuklaganj Bridge Kanpur	1	0.265	0.134	0.246	0.103	0.255	0.111	0.272	0.147	0.265	0.136	0.241	0.101
	2	0.413	0.238	0.361	0.184	0.363	0.196	0.412	0.243	0.396	0.217	0.34	0.181
	3	0.575	0.349	0.494	0.273	0.489	0.291	0.583	0.372	0.531	0.308	0.458	0.26
Bridge at Ansi, Fatehpur	1	0.528	0.244	0.522	0.237	0.515	0.233	0.541	0.262	0.536	0.254	0.514	0.232
	2	0.875	0.459	0.851	0.422	0.831	0.415	0.907	0.49	0.844	0.431	0.821	0.405
	3	1.247	0.691	1.215	0.618	1.165	0.612	1.269	0.697	1.219	0.656	1.158	0.61
River Gomti in Varanasi	1	0.71	0.243	1.455	0.411	0.931	0.31	0.768	0.356	0.701	0.248	0.694	0.231
	2	1.07	0.444	1.668	0.579	1.408	0.53	1.076	0.475	1.02	0.45	0.968	0.418
	3	1.422	0.669	1.874	0.757	1.711	0.732	1.403	0.785	1.346	0.65	1.219	0.605
Rajwari	1	0.532	0.299	0.537	0.288	0.553	0.306	0.539	0.32	0.524	0.292	0.513	0.284
	2	0.726	0.444	0.692	0.402	0.736	0.436	0.732	0.446	0.711	0.42	0.692	0.401
	3	0.909	0.579	0.91	0.551	0.914	0.569	0.914	0.593	0.901	0.563	0.872	0.538

The CEEMDAN-AdaBoost-BiLSTM model demonstrates up to 15.517%, 12.290% and 11.111% reductions over CEEMDAN-BiLSTM for one, two, and three-hour ahead predictions respectively for RMSE, across all stations. Similarly, for MAE reductions of up to 21.519%, 13.803%, and 18.182% across all stations is noticed for one, two, and three-hour ahead predictions respectively. This suggests that using AdaBoost can enhance the performance when compared with using only an individual BiLSTM model. Training multiple BiLSTM models in conjunction with the Adaboost algorithm involves a dynamic adjustment of model weights based on prediction errors. This collaborative approach serves to enhance forecasting outcome and robustness.

However, using the same approach to model all the components might not be optimal. The different IMF components are likely to exhibit distinct characteristics. High and medium-frequency IMFs, marked by swift fluctuations and intricate patterns, can be accurately predicted with AdaBoost-BiLSTM. These IMFs signify short-term variations and exhibit high randomness. Conversely, low-frequency IMFs and residual components, primarily representing gradual trends or periodic elements, are more effectively predicted using standalone LSTM. The proposed CEEMDAN-AdaBoost-BiLSTM-LSTM model outperforms CEEMDAN-AdaBoost-BiLSTM with reductions of up to 27.491%, 20.375%, and 11.567% for one, two, and three-hour ahead predictions respectively for RMSE, across all stations. For MAE, the reductions up to 31.122%, 34.317%, and 21.323% are seen across one, two, and three-hour ahead predictions respectively. This approach acknowledges the variability among components and emphasizes the importance of adapting prediction techniques to the individual characteristics of each component in order to produce more accurate results. The proposed approach has consistently provided the best forecast across all forecast horizons and all stations, thus demonstrating its superiority. It can be observed that, as the prediction time step increases, the predictive performance of all models gradually declines, demonstrating an increasing accumulation of errors in multi-step forecasting. This phenomena correctly depicts that predicting further into the future gets more difficult as the time step lengthens. However, improved outcomes are still obtained for our proposed CEEMDAN-AdaBoost-BiLSTM-LSTM model.

**Table 2:** Prediction results on test data for decomposition based hybrid models.

Station	Hour	CEEMDAN-ANN		CEEMDAN-LSTM		CEEMDAN-BiLSTM		CEEMDAN-AdaBoost-BiLSTM		CEEMDAN-AdaBoost-BiLSTM-LSTM	
		RMSE	MAE	RMSE	MAE	RMSE	MAE	RMSE	MAE	RMSE	MAE
Barrage at Narora	1	0.098	0.058	0.092	0.055	0.091	0.055	0.09	0.05	**0.09**	**0.047**
	2	0.151	0.09	0.144	0.077	0.131	0.076	0.128	0.076	**0.122**	**0.062**
	3	0.195	0.118	0.185	0.104	0.182	0.1	0.164	0.092	**0.157**	**0.085**
Kachla Ghat, Badaun	1	0.804	0.298	0.771	0.272	0.744	0.26	0.695	0.248	**0.669**	**0.233**
	2	0.943	0.366	0.914	0.338	0.897	0.332	0.896	0.33	**0.854**	**0.328**
	3	1.03	0.422	1.003	0.411	0.987	0.407	0.966	0.378	**0.939**	**0.363**
Ghatiya ghat, Farrukhabad	1	0.118	0.065	0.117	0.046	0.108	0.042	0.100	0.041	**0.099**	**0.035**
	2	0.131	0.066	0.121	0.051	0.121	0.049	0.12	0.049	**0.119**	**0.047**
	3	0.175	0.085	0.164	0.082	0.16	0.077	0.150	0.063	**0.149**	**0.062**
Bridge at Bithoor	1	0.189	0.116	0.132	0.089	0.126	0.079	0.115	0.062	**0.097**	**0.049**
	2	0.225	0.125	0.214	0.124	0.209	0.118	0.189	0.104	**0.145**	**0.079**
	3	0.311	0.182	0.288	0.161	0.272	0.158	0.253	0.142	**0.243**	**0.139**
Ganga Barrage, Kanpur	1	0.412	0.232	0.335	0.225	0.293	0.219	0.291	0.196	**0.211**	**0.135**
	2	0.487	0.364	0.457	0.327	0.407	0.295	0.373	0.271	**0.297**	**0.178**
	3	0.525	0.414	0.513	0.362	0.439	0.325	0.406	0.272	**0.361**	**0.214**
Bathing Ghat, Kanpur	1	0.52	0.243	0.501	0.241	0.454	0.218	0.453	0.215	**0.425**	**0.213**
	2	0.871	0.481	0.751	0.37	0.716	0.355	0.628	0.306	**0.602**	**0.299**
	3	0.78	0.405	0.772	0.407	0.771	0.405	0.76	0.377	**0.707**	**0.356**
Shuklaganj Bridge, Kanpur	1	0.157	0.084	0.138	0.081	0.138	0.074	0.138	0.062	**0.127**	**0.054**
	2	0.201	0.124	0.198	0.114	0.19	0.107	0.186	0.094	**0.176**	**0.088**
	3	0.315	0.209	0.283	0.206	0.282	0.158	0.268	0.149	**0.237**	**0.127**
Bridge at Ansi, Fatehpur	1	0.334	0.175	0.299	0.163	0.298	0.161	0.279	0.143	**0.279**	**0.137**
	2	0.6	0.38	0.554	0.288	0.508	0.288	0.492	0.284	**0.473**	**0.253**
	3	0.822	0.52	0.803	0.483	0.801	0.485	0.712	0.426	**0.686**	**0.388**
River Gomti in Varanasi	1	0.449	0.213	0.407	0.155	0.406	0.154	0.343	0.148	**0.337**	**0.132**
	2	0.668	0.339	0.621	0.281	0.598	0.258	0.571	0.255	**0.544**	**0.253**
	3	0.844	0.5	0.826	0.451	0.807	0.417	0.787	0.407	**0.727**	**0.358**
Rajwari	1	0.274	0.166	0.274	0.161	0.26	0.149	0.258	0.138	**0.256**	**0.131**
	2	0.41	0.285	0.377	0.219	0.367	0.218	0.361	0.205	**0.354**	**0.202**
	3	0.472	0.306	0.463	0.31	0.46	0.306	0.441	0.27	**0.427**	**0.248**

Although decomposing the original data is an additional computational step in the modeling process, it significantly enhances prediction accuracy. The simple and less complex decomposition-based models apply the same technique to forecast all the IMFs without considering their characteristics. While this approach may seem straightforward, it comes at the expense of predictive accuracy. The proposed CEEMDAN-AdaBoost-BiLSTM-LSTM model introduces an additional step of computing the zero-crossing rate for each IMF, aiding in selecting the appropriate forecasting model (AdaBoost-BiLSTM or LSTM) for each IMF, thereby improving accuracy. This computation incurs only a minimal increase in computation overhead and execution time, taking approximately 0.0114 seconds (example of Bithoor dataset). The application of AdaBoost-BiLSTM to the complex high and medium frequency IMFs further enhances predictive performance through ensemble learning techniques, making it a useful approach in critical real-world applications like water quality monitoring and forecasting.

Diebold-Mariano (DM) test^31^ and t-test are commonly used statistical tests used to compare the performance of models relative to each other^32–35^. DM test with the squared error as the loss function is used to compare the proposed forecasting model with ten alternative models. The DM statistics obtained are compiled in Supplementary Table S5. First, the magnitude of the DM value shows that the proposed model has a significant advantage over the other models in terms of forecasting accuracy. Furthermore, the computed p-values play a critical role in determining the statistical significance of the observed differences. Notably, each p-value was found below the 0.05 significance level. This suggests that the forecast accuracy of the proposed model differs statistically by a significant amount from that of the alternative models. Additionally, we conducted two-sided t-tests using results from seven different executions using different random seeding to evaluate the significance of performance variations among different datasets, as outlined in Supplementary Table S6. Notably, the p-values consistently remained below the 0.05 significance level, indicating that the proposed model’s RMSE scores significantly outperformed those of the benchmark models across all datasets.

Considering the dataset for the Bithoor station, the average runtimes for the individual models are as follows: 7.35 seconds for LR, 15.33 seconds for SVR, 10.32 seconds for RF, 90.20 seconds for ANN, 117.23 seconds for LSTM and 126.11 seconds for BiLSTM. The models with decomposition as a pre-processing step have the following average runtimes: 910.02 seconds for CEEMDAN-ANN, 1233.71 seconds for CEEMDAN-LSTM, 1254.75 seconds for CEEMDAN-BiLSTM, 3143.23 seconds for CEEMDAN-AdaBoost-BiLSTM and 1656.70 seconds for CEEMDAN-AdaBoost-BiLSTM-LSTM. The data decomposition pre-processing step increases the runtime of the hybrid models. Out of all the models, CEEMDAN-AdaBoost-BiLSTM takes the longest to run. It is worth noting that while the CEEMDAN-AdaBoost-BiLSTM model may show comparable accuracy levels to our proposed CEEMDAN-AdaBoost-BiLSTM-LSTM model in a few isolated cases, it does so at the cost of increased execution time. This is because the former model applies AdaBoost-BiLSTM to all decomposed components, whereas our proposed model applies AdaBoost-BiLSTM only to the high and medium frequency IMFs. By applying AdaBoost only to specific components, our model significantly reduces the execution time. While simpler models may exhibit close accuracy in a few isolated instances, our proposed model consistently demonstrates superior forecasting accuracy across all cases.

## Conclusion

This study examines the use of a novel approach combining the CEEMDAN approach in conjunction with AdaBoost and deep-learning for short-term and multi-step forecasting of the DO levels in river Ganga. The experiments demonstrate that:

1. A more precise and superior prediction result is achieved by taking into account the unique qualities of each IMF component and using a variety of prediction techniques catered to these components. Here, the AdaBoost-BiLSTM model is used to predict the high and medium frequency IMFs with complex non-linear patterns and standalone LSTM model is used for the low-frequency IMFs and residual that display reasonably simple and smooth patterns. The proposed approach outperforms all the models that used the same technique to forecast each component.
2. The proposed CEEMDAN-AdaBoost-BiLSTM-LSTM model outperforms the CEEMDAN-AdaBoost-BiLSTM with reductions in RMSE by up to 27.491%, 23.280%, and 11.567% and the reductions in MAE by up to 31.122%, 34.317%, and 21.323% for 1, 2, and 3-hour ahead predictions respectively. Compared with CEEMDAN-BiLSTM, the proposed model demonstrates up to 27.986%, 30.622% and 17.767% reductions for RMSE, and up to 38.356%, 39.661%, and 34.154% reductions in MAE for 1, 2, and 3-hour ahead predictions respectively. Results of Diebold-Mariano and t-test suggests statistically significant difference in forecasts between our proposed and the models used in comparison.
3. The proposed CEEMDAN-AdaBoost-BiLSTM-LSTM model is comparatively more computationally efficient than CEEMDAN-AdaBoost-BiLSTM model. The proposed model applies AdaBoost-BiLSTM only to the high frequency and medium frequency IMFs and employs a standalone LSTM model to the low frequency IMFs and residual. This takes lesser time to execute than CEEMDAN-AdaBoost-BiLSTM which applies AdaBoost-BiLSTM to all the IMFs and residual. However, the proposed model requires slightly longer processing time compared to the other simpler models. The future research will focus on integrating parallelization techniques for decreasing the computational time of the model making it more practically applicable while optimizing the performance.

### Supplementary Information

Supplementary Information.

## Data Availability

Data and materials are available from the corresponding author upon request.
