# Supplementary material for: Multi-step forecasting of dissolved oxygen in River Ganga based on CEEMDAN-AdaBoost-BiLSTM-LSTM model
Source: Sci Rep. 2024 May 16;14:11199. doi: 10.1038/s41598-024-61910-w (PMC11099056; doi:10.1038/s41598-024-61910-w)
Supplement: Supplementary file 1 — Supplementary Information. [file 41598_2024_61910_MOESM1_ESM.pdf]

Multi-step forecasting of dissolved oxygen in River Ganga based on CEEMDAN-AdaBoost-BiLSTM-LSTM model

Neha Pant[1], Durga Toshniwal[1, *], and Bhola Ram Gurjar[2]

[1]Indian Institute of Technology Roorkee, Computer Science and Engineering, Roorkee, 247667, India
[2]Indian Institute of Technology Roorkee, Civil Engineering, Roorkee, 247667, India
[*]durga.toshniwal@cs.iitr.ac.in

SUPPLEMENTARY INFORMATION

1. Data Collection
2. Optimal selection of hyperparameters
3. Methodolgy: Predicting CEEMDAN Components
4. Performance Evaluation
5. Diebold-Mariano test
6. Two-sided t-test
7. Performance Evaluation
8. Supplementary Table S1: Locations of River Ganga along with the latitude, longitude, and total number of records.
9. Supplementary Table S2: Significant lag selected for each component for datasets of all stations.
10. Supplementary Table S3: List of hyperparameters and their search space considered while building forecasting models.
11. Supplementary Table S4: Zero crossing rates of each component for datasets of all stations.
12. Supplementary Table S5: Diebold Mariano test statistics of the models considered in the study.
13. Supplementary Table S6: p-values obtained from the two-sided t-test for comparing the proposed CEEMDAN-AdaBoost-BiLSTM-LSTM model with the other models.

Data Collection

The data utilized in our study is obtained from the Uttar Pradesh Pollution Control Board (UPPCB), India. This dataset comprises real-time measurements of Dissolved Oxygen (DO) levels obtained through the Real-time Water Quality Monitoring System (RTWQMS) installed at multiple locations along the River Ganga, its principal tributaries, and drains. This comprehensive dataset offers insights into the fluctuation of DO levels in the River Ganga. The details of the data at the studied stations is indicated in Suppementary Table S1.

Station	Type	Latitude	Longitude	Records
Barrage at Narora	River	28°11’25.3"	78°23’43.2"	31569
Kachla Ghat, Badaun	River	27°55’51.8"	78°51’19.0"	24420
Ghatiya ghat, Farrukhabad	River	27°23’92.4"	79°37’62.5"	31970
Bridge at Bithoor	River	26°36’00.6"	80°16’26.8"	33526
Ganga Barrage, Kanpur	River	26°30’28.9"	80°18’59.5"	35225
Bathing Ghat, Kanpur	River	26°22’34.1"	80°29’32.9"	35760
Shuklaganj Bridge, Kanpur	River	26°27’42.01"	80°12’34.73"	33684
Bridge at Ansi, Fatehpur	River	26°03’17.5"	80°54’34.3"	35094
River Gomti in Varanasi	Tributary	25°30’24.6"	83°08’27.5"	34938
Rajwari	River	25°03’21.72"	83°11’57.6"	34911

Supplementary Table S1. Locations of River Ganga along with the latitude, longitude, and total number of records.

Station	Barrage at Narora	Kachla Ghat, Badaun	Ghatiya Ghat, Farukkhabad	Bithoor	Ganga Barrage, Kanpur	Bathing Ghat, Kanpur	Shuklaganj Bridge, Kanpur	Bridge at Ansi, Fatehpur	River Gomti in Varanasi	Rajwari
Original	1-4	1-3	1-4	1-3	1-2	1-2	1-4	1-4	1-4	1-4
IMF 1	1	1-5	1	1	1-2	1-7	1-4	1	1	1
IMF 2	1-4	1-4	1-4	1-10	1-5	1-4	1-5	1-10	1-10	1-4
IMF 3	1-4	1-4	1-10	1-9	1-6	1-6	1-9	1-9	1-10	1-6
IMF 4	1-7	1-8	1-4	1-3	1-6	1-8	1-6	1-7	1-5	1-7
IMF 5	1-5	1-8	1-5	1-5	1-7	1-7	1-6	1-3	1-7	1-5
IMF 6	1-6	1-5	1-6	1-7	1-10	1-9	1-10	1-8	1-10	1-6
IMF 7	1-10	1-6	1-8	1-7	1-9	1-10	1-5	1-10	1-4	1-7
IMF 8	1-4	1-8	1-5	1-9	1-6	1-10	1-6	1-8	1-8	1-5
IMF 9	1-6	1-10	1-8	1-9	1-9	1-6	1-9	1-6	1	1-6
IMF 10	1-6	1-10	1-10	1-7	1-9	1-9	1	1	1	1-9
IMF 11	1-9	1-5	1	1-6	1-9	1	1	1	1	1-10
IMF 12	1	1-10	1	1	1	1	1	1	1	1
IMF 13	1	1-9	1	NA	1	1	1	1	1	1
IMF 14	NA	1	1	NA	NA	NA	NA	NA	NA	1
IMF 15	NA	1	NA	NA	NA	NA	NA	NA	NA	1
IMF 16	NA	1	NA	NA	NA	NA	NA	NA	NA	NA
residual	1	1	1	1	1	1	1	1	1	1

Supplementary Table S2. Significant lag selected for each component for datasets of all stations.

Optimal selection of hyperparameters

While implementing the proposed and benchmark models we have recognized the importance of capturing temporal dependencies in our data and employed autocorrelation function (ACF) and partial autocorrelation function (PACF) analysis to select the optimal number of lags (Suppementary Table S2) which is used by

all the models (with decomposition and without decomposition). For the learning rate, we have tested our models on 0.01, 0.001, and 0.0001, with 0.001 consistently yielding the best results across all datasets. Similarly, for batch size, we explored values of 32, 64, and 128, with 64 emerging as the optimal choice. Regarding activation functions, we compared ReLU and tanh functions, finding ReLU to be the most effective in capturing the non-linear relationships present in our data. In the end we have seven independent executions using different random seeding for each model with the chosen optimal hyperparameters. The Suppementary Table S3 represent the search space for the hyperparameters of different models considered in the study.

**Hyperparameters**	**Search Space**
**Support Vector Regression**	
kernel	[linear, poly, rbf, sigmoid]
epsilon	[0.001, 0.01, 0.1, 1]
C	[0.1, 1, 10, 100]
**Random Forest**	
n_estimators	[1-100]
max_depth	[1-50]
max_features	[1-10]
**ANN, LSTM, BiLSTM**	
Learning Rate	0.01, 0.001, 0.0001
Batch Size	32, 64, 128
Activation Function	Tanh, ReLU
Optimizer	Adam, RMSprop
Loss Function	mse, mae
epochs	[5-100]

Supplementary Table S3. List of hyperparameters and their search space considered while building forecasting models.

Methodolgy: Predicting CEEMDAN Components

CEEMDAN is a data decomposition technique that decomposes the data into multiple subseries called IMFs and residual. The IMFs encompass a spectrum of characteristics, extending from high-frequency to low-frequency, with each IMF exhibiting its unique inherent properties. Instead of applying a uniform model across all IMFs, we employ different model for the IMFs based on their characteristics. For this, first we calculate zero-crossing rate to check the fluctuation frequency of the data in each IMF and then categorize them into high, medium and low frequency components. The zero-crossing rate can be calculated as follows:

$$zero\ crossing\ rate = \frac{N}{T} \tag{1}$$

where N is the total number of zero crossings and T is the duration of the time interval. If zero-crossing rate of IMF ≥ 0.01 then the IMF is categorized as high/medium frequency IMF otherwise the IMF is categorized as a low frequency IMF. The zero-crossing rate of the IMFs obtained is given in Supplementary Table S4. We use ACF and PACF to find the significant lags for each IMF and residual. Then, AdaBoost-BiLSTM model is applied to predict the high and medium-frequency IMFs, which are characterized by

complex patterns and frequent changes over time. On the other hand, the standalone LSTM model is used to predict the low-frequency IMFs and residual components.

Station	Barrage at Narora	Kachla Ghat, Badaun	Ghatiya Ghat, Farukkhabad	Bithoor	Ganga Barrage, Kanpur	Bathing Ghat, Kanpur	Shuklaganj Bridge, Kanpur	Bridge at Ansi, Fatehpur	River Gomti in Varanasi	Rajwari
IMF 1	0.6736	0.6597	0.6860	0.6725	0.6774	0.6426	0.6765	0.6526	0.6601	0.6902
IMF 2	0.4828	0.5628	0.5210	0.4626	0.4237	0.4882	0.4550	0.4088	0.4369	0.4315
IMF 3	0.1819	0.2332	0.1693	0.1418	0.1908	0.1969	0.1814	0.1399	0.1453	0.2055
IMF 4	0.0945	0.1284	0.0958	0.0847	0.0954	0.1104	0.0939	0.0855	0.0868	0.1124
IMF 5	0.0625	0.0815	0.0621	0.0581	0.0616	0.0654	0.0624	0.0540	0.0571	0.0755
IMF 6	0.0321	0.0546	0.0307	0.0280	0.0335	0.0365	0.0331	0.0287	0.0297	0.0475
IMF 7	0.0164	0.0354	0.0152	0.0142	0.0172	0.0192	0.0167	0.0150	0.0155	0.0280
IMF 8	0.0075	0.0239	0.0075	0.0063	0.0090	0.0092	0.0082	0.0077	0.0080	0.0169
IMF 9	0.0035	0.0162	0.0036	0.0028	0.0041	0.0042	0.0038	0.0032	0.0043	0.0097
IMF 10	0.0014	0.0097	0.0018	0.0013	0.0017	0.0021	0.0016	0.0015	0.0018	0.0046
IMF 11	0.0007	0.0060	0.0007	0.0004	0.0007	0.0008	0.0005	0.0005	0.0008	0.0023
IMF 12	0.0002	0.0034	0.0002	0.0001	0.0003	0.0003	0.0002	0.0002	0.0002	0.0010
IMF 13	0.0001	0.0015	0.0001	NA	0.0001	0.0001	5.0684E-05	7.6026E-05	0.0001	0.0004
IMF 14	NA	0.0006	7.6026E-05	NA	NA	NA	NA	NA	NA	0.0001
IMF 15	NA	0.0001	NA	NA	NA	NA	NA	NA	NA	5.0684E-05
IMF 16	NA	7.6026E-05	NA	NA	NA	NA	NA	NA	NA	NA
residual	0.0	0.0	0.0	0.0	0.0	0.0	0.0	0.0	0.0	0.0

Supplementary Table S4. Zero crossing rates of each component for datasets of all stations.

Performance Evaluation

The methodology used to evaluate the performance of the proposed CEEMDAN-AdaBoost-BiLSTM-LSTM model across the ten stations along the river Ganga involves comparing its forecasts with the actual DO levels. This is done by calculating the performance metrics such as Root Mean Square Error (RMSE), Mean Absolute Error (MAE), Mean Absolute Percentage Error (MAPE) and Coefficient of Determination (R^2).

1. RMSE is calculated as the square root of the average of squared errors and can be helpful in understanding the influence of outliers in the model performance.
2. MAE is a scale independent measure and can be calculated as the average of the absolute errors.
3. MAPE is used to determine the accuracy of the forecasting model by calculating the average of the absolute percentage error of each item in a dataset. MAPE values can be easily interpreted and can be used to compare forecast accuracy across multiple data sets.
4. R^2 measures the fraction of the variability in the outcome variable that can be accounted for by the predictor variable.

Smaller values for RMSE, MAE and MAPE and higher values of R^2 indicate better performance. We have used Python's sklearn library's metrics module for calculating the RMSE, MAE and R^2. We have written our own Python code for calculating MAPE.

Further, Diebold Mariano test is conducted to assess the statistical significance differences in forecast accuracy between the proposed CEEMDAN-AdaBoost-BiLSTM-LSTM model and other models used in comparison. We have used the dm_test() function from the dieboldmariano Python package to conduct the Diebold Mariano tests. Additionally, two-sided t-test further supports that there is a statistically significant difference in RMSE values between the proposed and compared models. We have used the ttest_rel() function from the Python's SciPy stats package to conduct the two-sided t-test.

Diebold-Mariano test

The Diebold-Mariano test provides a quantitative assessment of whether the differences in forecast accuracy between the proposed model and alternative approaches are statistically significant or merely due to random chance. When comparing the proposed CEEMDAN-AdaBoost-BiLSTM-LSTM model to alternative models without decomposition, the Diebold-Mariano test helps assess the impact of incorporating CEEMDAN decomposition, along with AdaBoost-BiLSTM and LSTM models in affecting the forecast accuracy. When comparing the proposed CEEMDAN-AdaBoost-BiLSTM-LSTM model to alternative models with decomposition, the Diebold-Mariano test helps assess the impact of utilizing a tailored prediction technique based on the distinctive features of each component (IMFs and residual) on improving the forecast accuracy. The proposed model significantly outperforms the models with and without decomposition. Consistently statistically significant test results (Supplementary Table S5) across various station datasets and forecast horizons strengthen the evidence for the proposed model's superiority, reliability and robustness.

Station	Hours	LR		SVR		RF		ANN		LSTM		BiLSTM		CEEMDAN ANN		CEEMDAN LSTM		CEEMDAN BiLSTM		CEEMDAN AdaBoost-BiLSTM	
		DM	p value	DM	p value	DM	p value	DM	p value	DM	p value	DM	p value	DM	p value	DM	p value	DM	p value	DM	p value
Barrage at Narora	1	20.55	6.32E-92	17.65	1.21E-68	19.20	9.91E-81	25.22	4.87E-136	19.90	2.01E-86	19.84	6.37E-86	4.80	1.65E-6	3.92	8.96E-5	6.35	2.20E-10	4.25	2.13E-5
	2	23.47	1.47E-118	20.84	2.15E-94	23.29	7.44E-117	24.90	9.03E-133	23.90	9.62E-123	23.42	4.32E-118	13.64	5.57E-42	9.50	2.48E-21	8.25	1.73E-16	6.79	1.16E-11
	3	27.26	7.52E-158	24.08	1.53E-124	26.77	2.02E-152	29.76	1.68E-186	28.40	1.16E-170	26.11	2.37E-145	15.35	1.42E-52	12.00	6.32E-33	10.56	6.46E-26	5.44	5.48E-8
Kachla Ghat, Badaun	1	2.79	5.337E-3	3.63	2.824E-4	3.71	2.967E-4	2.87	4.156E-3	2.52	1.159E-2	0.72	4.735E-1	4.85	1.24E-6	2.89	3.92E-3	3.06	2.246E-3	2.87	4.166E-3
	2	4.19	2.79E-5	2.75	5.903E-3	4.35	1.35E-5	3.80	1.458E-4	4.11	3.98E-5	2.40	1.639E-2	3.82	1.366E-4	2.57	1.019E-2	2.41	1.606E-2	2.74	6.065E-3
	3	3.34	8.417E-4	2.65	8.012E-3	4.30	1.69E-5	4.45	8.71E-6	3.67	2.416E-4	2.18	2.899E-2	3.55	3.937E-4	3.07	2.151E-3	2.42	1.541E-2	2.04	3.497E-2
Ghatiya ghat, Farrukhabad	1	5.31	1.15E-7	4.99	6.02E-7	4.57	5.05E-6	5.93	3.08E-9	7.39	1.56E-13	4.95	7.54E-7	1.99	3.913E-2	2.51	1.195E-2	2.03	3.429E-2	2.27	2.898E-2
	2	7.55	4.68E-14	9.32	1.39E-20	9.08	1.35E-19	12.21	4.57E-34	11.55	1.12E-30	8.91	6.09E-19	3.13	1.731E-3	2.23	4.35E-2	2.63	3.17E-2	2.35	3.251E-2
	3	12.07	2.72E-33	10.58	5.31E-26	11.62	5.34E-31	14.04	2.37E-44	17.68	6.99E-69	11.27	2.67E-29	3.26	1.118E-3	2.16	3.112E-2	2.51	1.316E-2	2.04	4.65E-2
Bridge at Bithoor	1	14.05	1.95E-44	10.53	8.40E-26	12.04	3.75E-33	15.01	2.29E-50	13.94	8.96E-44	14.38	1.95E-46	10.76	7.67E-27	7.22	5.53E-13	8.74	2.67E-18	10.92	1.40E-27
	2	8.53	1.68E-17	6.78	1.27E-11	9.19	4.74E-20	12.78	4.37E-37	10.00	1.91E-23	8.08	7.23E-16	12.01	5.04E-33	11.35	1.16E-29	10.73	1.00E-26	12.78	3.93E-37
	3	17.56	5.20E-68	15.68	9.86E-55	20.09	5.60E-88	19.78	1.95E-85	17.60	2.62E-68	17.00	6.70E-64	16.38	1.62E-59	9.92	4.25E-23	7.65	2.16E-14	2.41	1.577E-2
Ganga Barrage, Kanpur	1	16.86	6.20E-63	15.11	5.22E-51	15.70	7.38E-55	17.02	4.76E-64	16.24	1.53E-58	16.12	9.56E-58	32.57	3.99E-221	29.42	1.91E-182	26.75	2.91E-152	9.21	4.04E-20
	2	18.63	3.57E-76	15.62	2.26E-54	15.95	1.45E-56	19.06	1.54E-79	19.00	4.37E-79	16.71	7.40E-62	45.42	0.0	43.46	0.0	39.04	4.028e-310	24.86	2.12E-132
	3	23.85	2.64E-122	19.22	7.73E-81	19.54	1.95E-83	24.31	8.69E-127	22.02	6.74E-105	21.29	2.49E-98	41.65	0.0	35.74	3.57E-263	21.75	1.81E-102	12.48	1.74E-35
Bathing Ghat, Kanpur	1	8.45	3.42E-17	15.01	2.30E-50	11.90	1.97E-32	8.99	3.05E-19	8.58	1.05E-17	7.94	2.23E-15	2.30	2.15E-2	6.17	7.20E-10	3.45	5.535E-4	3.92	9.04E-5
	2	8.66	5.52E-18	15.74	3.76E-55	14.99	2.90E-50	9.18	5.18E-20	9.76	2.21E-22	9.30	1.63E-20	16.88	4.82E-63	11.98	8.06E-33	11.61	5.81E-31	3.58	3.413E-4
	3	11.68	2.46E-31	17.11	1.09E-64	16.38	1.56E-59	11.97	8.88E-33	13.51	3.37E-41	11.48	2.66E-30	5.71	1.19E-8	6.93	4.47E-12	6.28	3.53E-10	6.53	6.86E-11
Shuklaganj Bridge, Kanpur	1	19.99	3.53E-87	9.64	6.76E-22	10.73	1.00E-26	19.72	6.58E-85	18.01	2.22E-71	11.19	6.76E-29	11.88	2.56E-32	10.69	1.59E-26	10.24	1.71E-24	5.42	6.21E-8
	2	17.73	2.99E-69	8.25	1.83E-16	11.88	2.37E-32	18.36	4.39E-74	14.12	8.25E-45	11.03	4.06E-28	6.08	1.24E-9	6.24	4.66E-10	4.59	4.40E-6	4.52	6.23E-6
	3	26.71	8.18E-152	11.19	7.13E-29	17.15	5.48E-65	28.83	1.66E-175	20.86	1.44E-94	14.65	4.08E-48	19.68	1.43E-84	16.20	2.81E-58	16.19	3.35E-58	10.68	1.75E-26
Bridge at Ansi, Fatehpur	1	15.62	2.34E-54	11.36	9.86E-30	12.23	3.83E-34	16.28	8.28E-59	16.50	2.33E-60	13.33	3.67E-40	8.79	1.72E-18	6.23	5.01E-10	6.33	2.54E-10	6.08	1.21E-9
	2	14.31	5.70E-46	13.50	3.70E-41	13.90	1.62E-43	15.48	2.01E-53	14.43	9.56E-47	13.62	7.67E-42	13.87	2.40E-43	7.80	7.09E-15	4.67	3.06E-6	5.82	5.97E-9
	3	17.34	2.08E-66	15.99	7.83E-57	16.22	2.08E-58	18.22	5.44E-73	17.28	5.91E-66	16.29	6.68E-59	9.48	2.99E-21	8.58	1.09E-17	8.68	4.77E-18	6.41	1.48E-10
River Gomti, Varanasi	1	7.52	5.93E-14	13.46	6.00E-41	12.88	1.21E-37	8.94	4.54E-19	7.59	3.55E-14	6.98	3.18E-12	7.48	8.20E-14	3.21	1.341E-3	3.21	1.345E-3	2.09	1.046E-2
	2	9.28	2.13E-20	15.52	1.17E-53	16.40	1.10E-59	10.37	4.66E-25	9.18	5.05E-20	9.10	1.06E-19	6.07	1.32E-9	3.37	7.453E-4	2.75	5.977E-3	2.385	6.406E-3
	3	11.06	2.83E-28	17.17	3.91E-65	17.46	3.06E-67	11.63	4.58E-31	11.48	2.73E-30	13.37	2.13E-40	6.00	2.03E-9	3.75	1.764E-4	4.97	6.64E-7	2.51	1.222E-2
Rajwari	1	20.74	1.47E-93	19.61	4.77E-84	20.09	4.74E-88	21.57	6.94E-101	20.56	5.09E-92	19.98	4.25E-87	11.92	1.49E-32	13.79	7.26E-43	6.96	3.73E-12	3.66	2.503E-4
	2	23.57	1.43E-119	21.52	2.11E-100	23.51	6.26E-119	24.02	6.56E-124	23.26	1.37E-116	22.09	1.49E-105	21.26	4.35E-98	10.10	7.18E-24	6.94	4.29E-12	2.19	2.861E-2
	3	29.63	5.91E-185	26.72	6.07E-152	29.23	3.19E-180	30.37	8.77E-194	29.41	2.91E-182	28.17	5.55E-168	8.05	9.38E-16	9.87	7.07E-23	9.15	6.52E-20	3.51	4.505E-4

Supplementary Table S5. Diebold Mariano test statistics of the models considered in the study.

Two-sided t-test

The two-sided t-test, also known as the two-tailed t-test, is a statistical method employed to ascertain whether there exists a significant difference between the means of two independent groups. In this scenario,

the null hypothesis posits no significant difference between the RMSE values of the two models under comparison. A significance level of 0.05 is chosen. Notably, each p-value was found below the 0.05 significance level (Supplementary Table S6). This outcome indicates robust evidence supporting the alternative hypothesis, which asserts a substantial discrepancy in RMSE values between the two models. Consequently, the two models exhibit discernibly divergent predictive performance.

Station	Hours	RF	ANN	LSTM	BiLSTM	CEEMDAN ANN	CEEMDAN LSTM	CEEMDAN BiLSTM	CEEMDAN AdaBoost-BiLSTM
Narora	1	2.715e-08	5.426e-07	4.788e-06	5.688e-08	6.723e-03	1.859e-02	3.445e-02	4.868e-02
	2	8.473e-08	1.035e-07	1.249e-07	3.191e-07	9.317e-05	6.156e-04	1.046e-03	6.191e-03
	3	2.139e-08	3.638e-06	1.551e-06	7.322e-06	5.602e-04	1.401e-03	3.628e-03	7.769e-05
Kacha Ghat Badaun	1	5.357e-08	7.986e-05	5.573e-06	8.366e-08	3.415e-09	1.782e-06	8.249e-05	5.356e-05
	2	2.358e-04	3.749e-06	1.250e-07	1.109e-04	1.838e-04	4.759e-05	4.360e-04	3.441e-04
	3	1.321e-03	5.838e-03	1.356e-05	8.192e-04	5.838e-03	1.361e-03	4.468e-02	1.194e-03
Ghatiya Ghat, Farukkhabad	1	4.229e-06	1.914e-05	3.422e-04	1.864e-04	1.641e-02	2.508e-02	2.156e-02	3.163e-02
	2	2.989e-03	5.842e-04	3.221e-03	4.389e-03	7.460e-03	2.617e-02	3.520e-02	4.337e-02
	3	1.507e-05	4.327e-04	6.441e-06	1.796e-05	1.854e-02	3.652e-02	3.737e-02	2.445e-02
Bithoor	1	1.140e-06	1.114e-06	1.237e-06	2.481e-06	1.179e-05	4.965e-04	4.327e-04	1.128e-05
	2	2.062e-06	5.213e-06	4.928e-07	2.284e-06	1.347e-04	1.584e-04	1.127e-03	1.295e-03
	3	1.076e-06	4.530e-06	6.480e-06	4.660e-06	5.139e-03	1.809e-03	5.383e-03	2.170e-02
Ganga Barrage, Kanpur	1	2.997e-07	3.185e-06	3.675e-06	1.903e-06	5.784e-06	1.294e-04	1.673e-04	1.856e-04
	2	5.484e-05	4.443e-05	4.567e-05	5.037e-05	3.326e-04	4.741e-04	1.669e-03	7.910e-03
	3	3.784e-08	3.392e-08	1.289e-08	2.965e-08	2.517e-08	1.838e-07	3.231e-06	9.338e-05
Bathing Ghat, Kanpur	1	4.157e-08	1.408e-07	7.457e-08	9.120e-08	1.146e-04	2.944e-05	1.055e-03	3.882e-04
	2	1.719e-06	9.876e-08	6.730e-07	1.055e-07	1.657e-05	9.515e-06	3.937e-05	6.917e-04
	3	5.354e-07	9.457e-08	6.901e-07	1.002e-06	1.338e-04	3.984e-05	7.020e-05	1.290e-02
Shuklaganj Bridge , Kanpur	1	4.974e-07	3.939e-05	2.196e-05	3.947e-05	2.599e-03	5.127e-03	8.303e-03	2.583e-03
	2	6.207e-06	3.939e-05	2.196e-05	6.653e-08	1.338e-04	2.449e-02	2.178e-03	2.105e-03
	3	2.904e-05	8.956e-06	4.927e-06	1.970e-06	8.976e-04	2.398e-03	2.178e-03	1.495e-04
Bridge at Ansi, Fatehpur	1	7.017e-07	3.797e-07	9.732e-08	1.301e-06	4.118e-04	2.591e-02	2.733e-02	3.475e-02
	2	1.771e-07	4.709e-07	1.167e-07	1.066e-07	5.121e-05	6.374e-05	2.717e-03	2.007e-03
	3	2.366e-09	3.052e-09	5.269e-09	3.305e-07	2.988e-05	8.117e-05	7.259e-05	5.142e-04
River Gomti, Varanasi	1	1.537e-06	1.213e-07	2.470e-07	4.323e-07	5.716e-05	8.230e-05	5.940e-05	4.298e-02
	2	1.826e-07	1.403e-07	1.848e-07	6.287e-06	4.399e-04	4.162e-04	2.983e-03	1.604e-02
	3	1.874e-09	1.849e-08	7.047e-07	1.046e-05	5.790e-06	4.530e-06	1.305e-05	1.217e-08
Rajwari	1	7.319e-06	1.915e-05	2.266e-05	1.711e-05	3.269e-02	3.875e-02	4.865e-02	2.973e-02
	2	1.264e-08	9.352e-08	4.077e-10	4.889e-08	5.313e-04	5.923e-03	1.301e-02	7.555e-03
	3	3.648e-07	1.622e-08	4.456e-08	4.486e-07	3.786e-04	2.625e-04	1.310e-03	2.105e-03

Supplementary Table S6. p-values obtained from the two-sided t-test for comparing the proposed CEEMDAN-AdaBoost-BiLSTM-LSTM model with the other models.
